# Elderly and Patients with Large Breast Volume Have an Increased Risk of Seroma Formation after Mastectomy—Results of the SerMa Pilot Study

**DOI:** 10.3390/cancers15143606

**Published:** 2023-07-13

**Authors:** Melitta Beatrice Köpke, Carl Mathis Wild, Mariella Schneider, Nicole Pochert, Felicitas Schneider, Jacqueline Sagasser, Thorsten Kühn, Michael Untch, Christian Hinske, Matthias Reiger, Claudia Traidl-Hoffmann, Christian Dannecker, Udo Jeschke, Nina Ditsch

**Affiliations:** 1 Department of Gynecology and Obstetrics, University Hospital Augsburg, 86156 Augsburg, Germany; 2 Institute for Digital Medicine, University Augsburg, 86153 Neusäß, Germany; 3 Department for Environmental Medicine, Medical Faculty, University Augsburg, 86156 Augsburg, Germany; 4 Clinic for Gynaecology and Obstetrics, Filderklinik, Filderstadt-Bonlanden, 70794 Filderstadt, Germany; 5 Department of Gynecology and Obstetrics, University Hospital Ulm, 89070 Ulm, Germany; 6 Helios Clinic Berlin-Buch, Obstetrics and Gynaecology, 13125 Berlin, Germany

**Keywords:** breast cancer, seroma, mastectomy, implant

## Abstract

**Simple Summary:**

The pathophysiology behind seroma formation as a common postoperative complication after ablative procedures for breast cancer is poorly understood. Presented here is the first clinical evaluation of the SerMa (Seroma formations of the Mammary gland in breast cancer patients after mastectomy) pilot study, which investigates primarily possible immunological or inflammatory causes of seroma formation. Furthermore, clinicopathological correlations between seroma formation and tumor biology as well as lymph node involvement have been measured and showed a significant correlation with higher age and larger mastectomy specimen weight. Neither the number of lymph nodes removed or affected nor tumor biological characteristics like hormone receptor status showed a significant effect on seroma formation.

**Abstract:**

The collective of the SerMa pilot study included 100 cases of primary breast cancer or Carcinoma in situ who had undergone a mastectomy procedure with or without reconstruction of the breast using an implant or expander at Augsburg University Hospital between 12/2019 and 12/2022. The study aimed to investigate possible causes of seroma formation; reported here are the clinicopathological correlations between seroma formation and tumor biology and surgical procedures. Seroma occurred significantly more often in patients with older age (median patient age in cases with seroma was 73 years vs. 52 years without seroma; *p* < 0.001). In addition, patients with larger mastectomy specimen were significantly more likely to develop seroma (median ablation weight in cases with seroma 580 g vs. 330 g without seroma; *p* < 0.001). Other significant parameters for seroma formation were BMI (*p* = 0.005), grading (*p* = 0.015) and tumor size (*p* = 0.036). In addition, with insertion of implant or expander, a seroma occurred significantly less frequently (*p* < 0.001). In a binary logistic regression, age in particular was confirmed as a significant risk factor. In contrast, tumor biological characteristics, number of lymph nodes removed or affected showed no significant effect on seroma formation. The present study shows the need for patient education about the development of seroma in particular in older patients and patients with large breast volumes within the preoperative surgical clarification. These clinicopathological data support the previously published results hypothesizing that seroma formation is related to autoimmune/inflammatory processes and will be tested on a larger collective in the planned international multicenter SerMa study.

## 1. Introduction

Seroma formation is a common postoperative complication of breast cancer surgery, especially after mastectomy. Its incidence described in the literature varies strongly between 3% and 90% [1] and usually occurs within the first weeks after surgery. The pathophysiology behind seroma formation as an accumulation of fluid in a surgically newly formed cavity is poorly understood. It is still unclear whether the seroma fluid has to be seen as lymph-like fluid enriched by proteins or cells collected in the drained region [2] or as an exudate [3,4]. In addition, there is evidence that the duration of surgery or the use of electrocautery for hemostasis may promote seroma development [5,6]. The use and potential benefit of postoperative drainage of the surgical area also continues to be controversial: although frequently used in clinical practice, in the belief of incidence-reduction of seromas, a recent meta-analysis showed no disadvantage by omitting drainage with regard to seroma formation after mastectomy [7]. New prediction mechanisms are also under evaluation, for example an algorithm using artificial neural networks for the prediction of seroma development [8].

In order to address this issue and develop a better understanding of seroma formation after ablative surgery for breast cancer, the SerMa (Seroma formations of the Mammary gland in breast cancer patients after mastectomy) pilot study was initiated. This study investigates primarily possible immunological or inflammatory causes of seroma formation as the origin and composition of seroma must be understood at first, in order to develop strategies for its avoidance.

Our group has already been able to publish promising results regarding the cell content of the seroma fluids, suggesting a specific immunological response through certain T helper (Th) cell subpopulations [9]. Significantly higher numbers of Th2 and Th17 cells were found in the seroma fluid and peripheral blood of the same patients but not in peripheral blood of healthy controls. Furthermore, seroma formation after breast surgery was associated with an inflammatory immune response resulting in an increase of Th2/17 associated cytokines [10]. Based on the cytokine milieu, antigen presentation and expression of costimulatory molecules, activated Th cells differentiate into several functional classes [11]. From this the hypothesis can be derived whether Th2 and Th17 could be suitable biomarkers for a systemic immune event and therefore seroma formations.

Presented here is the first clinical evaluation of the SerMa pilot study. The correlation between clinicopathological parameters like tumor biology or lymph node involvement and the appearance of a seroma formation was examined, as this information is already available at time of diagnosis and may also have an influence on seroma formation.

## 2. Materials and Methods

The patient cohort of the monocentric SerMa pilot study included 100 primary breast cancer or carcinoma in situ cases (see Table 1), which was conducted at the Augsburg University Hospital between December 2019 and December 2022. A total of 14 patients presented with bilateral disease; each side is reported as a separate case. All patients had undergone a mastectomy procedure with or without implant or expander-based reconstruction of the breast. Exclusion criteria were recurrent breast cancer or metastatic disease at the time of diagnosis, as well as other carcinomas in medical history. To rule out a selection bias by possible altered wound healing processes or immunological shift in scarred tissue, patients with previous breast surgery (for benign or malignant disease) were excluded, as well.

All statistical analyses were performed with SPSS Statistics (IBM, Version 28.0.1.0). Using the Shapiro–Wilk test, the data was found to be non-normally distributed, therefore, appropriate tests in the absence of a normal distribution were used for further calculations. For testing the association between postoperative seroma formation and clinicopathological parameters, the Mann–Whitney U Test for graphical representation boxplots were used. To adjust for confounders, a binary logistic regression analysis with the significant parameters of the correlation analysis was performed. A value of *p* < 0.05 was considered statistically significant.

The study was performed according to the standards set in the Declaration of Helsinki 1975. The SerMa pilot study was approved by the ethics committee of the Ludwig-Maximilian-University Munich, Germany.

## 3. Results

### 3.1. Cohort Description

The age of the patients ranged from 30 to 91 years (median 63 years) and the median Body-Mass-Index (BMI) was 25.9. Table 1 provides an overview of the tumor characteristics.

The majority of patients had a NST (non-special type) carcinoma, 9 cases had precancerous lesions only (carcinoma in situ, CIS). The tumor was HR (hormone receptor (estrogen (ER) and/or progesterone (PR) receptor)) positive in 84 cases and 10 cases had a HER2-positive carcinoma. Locally advanced BC (≥pT3) was present in 22 cases, pathological nodal involvement of the axilla in 32 cases.

In total, 53 patient cases had received neoadjuvant chemotherapy, of which 16 cases achieved pathological complete remission (ypT0). In addition to mastectomy, almost all patients underwent axillary staging, the majority by sentinel lymph node biopsy (SLNB in 53 cases), with only 4 cases not undergoing axillary surgery (pNx).

Of all cases, 52 developed a seroma postoperatively, which had to be relieved by one or more punctures in 40 cases. In the majority of these cases (40.3%), only a single puncture was necessary.

**Table 1:** Clinical Overview—Patient and tumor characteristics. Since 100 cases are included, the percentages are identical to the absolute numbers.

	Median (Min.–Max.)
**Age at surgery (in years)**	63 (30–91)
**Body-Mass-Index (BMI)**	25.9 (18.9–56.6)
	n (number)
**Histopathological type**	
NST	68
Invasive lobular	16
Mucinous	3
Papillary	3
Other	1
CIS	9
**Hormone receptor status**	
HR+	84
ER−/PR−	16
**HER-2**	
Positive (IHC +++ or FISH pos.)	10
Negative (IHC 0)	67
Low (IHC + or ++, FISH neg.)	14
Not determined *	9
**Ki67**	
<20%	45
≥20%	46
Not determined *	9
**Tumor size**	
ypT0	16
pTis	8
pT1	30
pT2	24
pT3	17
pT4	5
**Axillary nodal status**	
pN0	64
pN1	20
pN2	10
pN3	2
pNx	4
**Grading**	
G1	13
G2	52
G3	33
No information available **	2

NST: Non-special type, CIS: Carcinoma in situ, HR: Hormone receptor, ER: Estrogen receptor. PR: Progesterone receptor, IHC: Immunohistochemistry, FISH: Fluorescence in situ hybridisation. * in case of carcinoma in situ, ** in case of external biopsy and diagnosing.

### 3.2. Correlation between Clinicopathological Parameters and Seroma Formation

**Figure 1:**
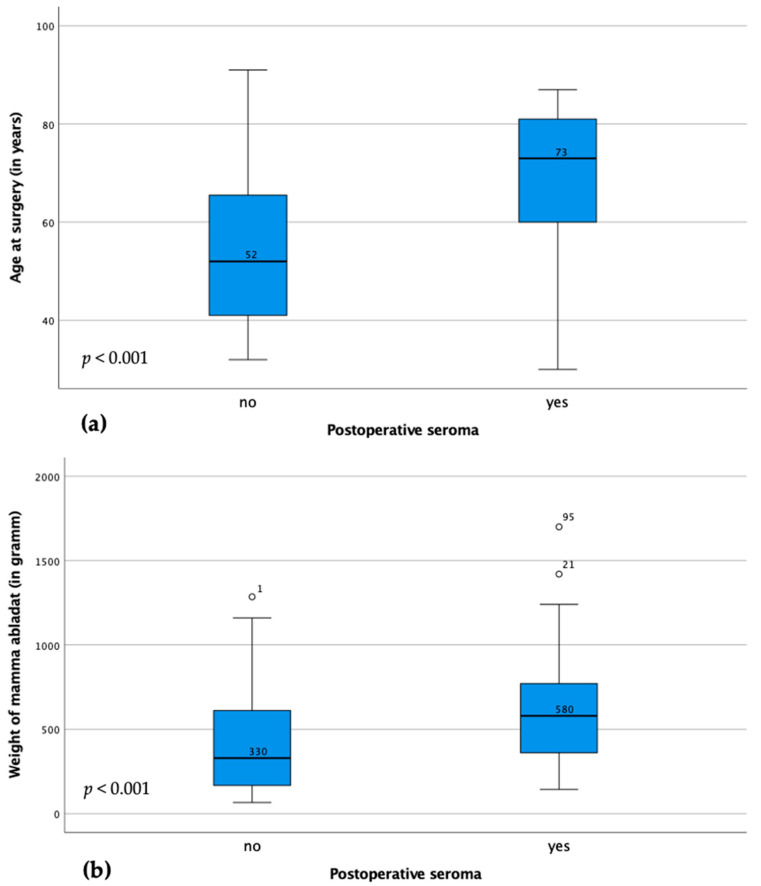
Illustration of the difference in age (**a**) and ablation weight (**b**) between patient cases with and without seroma. The boxes represent 25–75th percentiles, the error bars 5–95th percentiles. The line represents the median with indicated value. Circles and asterisks represent outliers (numbers represent case numbers).

Seromas are more common in elderly patients (median patient age in patient cases with seroma 73 years vs. 52 years without seroma; *p* < 0.001). In addition, patients with higher weight of the mastectomy specimen were significantly more likely to develop a seroma postoperatively (median mastectomy specimen weight in patient cases with seroma 580 g vs. 330 g without seroma; *p* < 0.001) (s. Figure 1). Other significant parameters were higher BMI (*p* = 0.005) and higher grading (*p* = 0.015). Patients with larger tumor size were also more likely to develop seroma postoperatively (*p* = 0.036). On the other hand, patients with immediate implant or expander reconstruction developed significantly less seroma in this cohort (*p* < 0.001), regardless of the size of the mastectomy specimen.

Tumor biological characteristics like the kind of histological subtype, the presence or absence of ER, PR or HER2 amplification showed no significant effect on seroma formation (*p* = 0.072, *p* = 0.844, *p* = 0.298, *p* = 0.494, respectively). Furthermore, neither the selected axillary intervention, the number of lymph nodes removed nor affected had a significant effect on seroma formation (see Table 2) and were independent of age at surgery.

### 3.3. Adjustment for Confounders by Binary Logistic Regression

In the binary logistic regression, the factors BMI, age at surgery, grading, mastectomy specimen weight, insertion of a foreign body and tumor stage (pT), as significant factors in the correlation analysis, were integrated (see Table 3). Interestingly, only age (*p* = 0.007) and grading (*p* = 0.019) remain significant factors. In contrast to the correlation analysis, higher grading appears protective in this analysis (OR = 0.318). Likewise, in contrast to the correlation, this analysis showed a trend for the insertion of a foreign body as a risk factor for seroma formation (OR = 1.025). For the ablation weight, there was a trend (OR = 1.002) toward increased seroma risk, consistent with the correlation analysis.

## 4. Discussion

The present study showed neither a significant influence of axillary nodal removal nor the tumor burden on seroma formation. Furthermore, this also applied to tumor biological characteristics. As there are very contradictory data from various studies, a possible association between lymph node removal, or involvement and seroma formation remains unclear [12]. Since other tumor biology characteristics (except grading) also showed no association with seroma development, these data are in line with the previously published results of our group, hypothesizing that seroma formation is rather related to autoimmune/inflammatory processes [9,10] than tumor biological characteristics.

The finding that age is a risk factor for development of a seroma is consistent with results from other studies [13]; nevertheless, the cause remains unclear. An association with hypertension, a common condition in the elderly, is an imaginable cause and has been seen in several studies [13,14,15]. A pathophysiological explanation discussed could be an increased continuous exudation in patients with hypertension and thus seroma formation [13]. Interestingly, the presence of hypertension was significantly associated with reconstruction failure after implant-based reconstruction in another study [16].

The presence of diabetes mellitus type II, another common comorbidity of the elderly, was also associated with increased seroma risk in other studies [17,18]. Due to low incidence in this cohort (only 13 cases were known to have diabetes), no valid statement can be made regarding a possible influence on seroma formation. Diabetes is also known to lead to altered immunological processes in the body and disrupt macrophage function for example [19]. Furthermore, epigenetic regulations of both immune and structural cells in wounds may influence cell phenotypes and healing in patients with type II diabetes [20]. However, with aging as a multifactorial process, the immune system is also known to change lifelong. For example, elderly people have a higher prevalence of autoimmunity and constitutive low-grade inflammation as an expression of age-associated immune dysfunction [21] and their immune system reacts slower to pathogens, tumor cells or foreign antigens.

What clinical consequence can be derived from this knowledge? In clinical practice, ablative surgery is often recommended for elderly, multimorbid patients in order to avoid possible postoperative radiation. With the results of this study, however, we must clearly move towards specifically pointing out to these patients an increased risk of seroma formation, which may also necessitate intensified further treatment like repeated relieving punctures, risk of infection and possibly necessary hospitalization. A feasible way of improving the patient education about individual risk factors for seroma formation could be to inform the patient within the preoperative surgical clarification. Providing a handout with a brief overview of how to potentially recognize a seroma early could also be helpful in encouraging patients to present early in case of seroma occurrence, thus avoiding uncertainty and suffering. Medical staff in general should also be trained to recognize possible seroma formation by clinical impression or anamnesis, in order to convey the affected to further help and increase compliance.

The second highly significant factor for seroma formation in this cohort was the weight of the breast specimen removed at surgery. It can be assumed that the resection of a larger surgical specimen also leaves a larger wound area in the majority of cases. This is represented not only by the length of the external visible scar, but also by the internal wound area. There have already been many attempts to close this “dead space” targeted with chemical (drugs) or mechanical means (surgically or by use of drainage) in order to reduce the seroma rate. It has shown to be advantageous in reducing the incidence and volume of seroma by obliterating the dead space through various flap apposition techniques [5,22]. Additionally, the use of fibrin glue can reduce seroma magnitude and duration; nevertheless, the data here is very inconsistent [23,24]. It is also possible that this may explain the positive impact of an implant- or expander-insert as the third highly significant factor in this cohort: by filling the dead space using a foreign body and by that modulating the physical-mechanical forces in the surgical area, altered postoperative processes for wound healing seem to occur, resulting in less frequent seroma formation. Another explanation for the possibly protective effect could be the use of a mesh for implant placement, an association already described in the literature [25]. Nevertheless, due to the small case number (insertion of implant or expander in only 33 cases), statistical significance cannot be equated with clinical significance and further investigations will have to confirm the observations made here. In particular, it cannot be derived that implant insertion should be recommended to elderly patients with planned mastectomy.

In this cohort, axillary tumor burden or surgery did not influence seroma formation. Contrary to earlier ideas that seroma fluid is identical to lymphatic fluid, it has now been shown that the seroma liquid has a higher protein content than lymph fluid and no fibrinogen is present, making coagulation impossible [26]. In this context, it seems quite understandable that the number of lymph nodes removed had no influence on development of seroma in the present study, despite a possible injury to the lymphatic vessels.

Within the binary logistic regression, only age and grading showed a significant influence on seroma formation (higher age as a risk factor, higher grading as protective). Since younger women with an often higher level of cosmetic awareness decide more frequently in favor of an implant reconstruction, it can be assumed that age as a strong confounder led to a distortion of the results and contrary statements regarding the influence of foreign body insertion on seroma formation. As in the correlation analysis, implant/expander insertion was classified as significantly highly protective, whereas the logistic regression showed a trend toward increased seroma formation risk with foreign body insertion. Furthermore, younger women in this cohort in particular had more aggressive tumors with higher grading, so again age may explain the putative protective effect of high grading in this analysis.

Several limitations exist in this study. The use and duration of electrocautery, a significant factor influencing seroma formation in other studies [5,6], was not the focus of the investigation here and therefore was not reported for this patient cohort. However, since this is a monocentric study with the same surgery methodology (with use of electrocautery for hemostasis) in all cases, this point seems negligible. In addition, the high seroma rate of 52% appears to be due to a selection bias as patients within the study were followed up more closely in domo than outside the study. Thus, seroma formation may have been detected earlier or more frequently. Compared to all patients who underwent mastectomy during this period at the university hospital Augsburg, the reported seroma rate was approximately 23%. A possible association with other comorbidities was not investigated in this collective, as this was not the primary objective of the SerMa pilot study. However, patients with known immunodeficiency were excluded; assuming that seroma formation is an (auto)immunological event, it seems comprehensible that the seroma rate is lower in the overall collective.

Consistently across all analyses, higher age has been shown to be a significant risk factor for seroma formation in this cohort. Since aging also leads to changes in the immune system, as shown above, the results presented here support the previously published analyses on a possible influence of an altered immune response on seroma development [9,10]. Further analyses including investigations of seroma fluid and blood, as well as tumor and microenvironment and especially possible changes with an aging immune system, are needed to answer the open immunological questions related to seroma formation. The planned international, multicenter SerMa study will have to show whether the knowledge gained in the pilot study can be transferred to a prospective design with a higher case number.

## 5. Conclusions

In summary, it can be deduced from the data of the SerMa pilot study that, in particular, older patients and patients with large breast volumes (and therefore expected large ablation) must be specifically informed about higher risk for postoperative seroma formation with possible protective and therapeutic approaches in the future. These approaches include a possibly adapted surgical therapy for older patients (e.g., favoring a breast-conserving therapy or active closing of the surgical cavity) and further immunological investigations within the planned multicenter SerMa study. A better understanding of the pathophysiology behind seroma formation could help identify patients at risk early, so new medicinal or surgical approaches can be developed for these patients to prevent seroma formation in future.

## Figures and Tables

**Table 2:** Comparison of surgical parameters and amount of removed and affected axillary lymph nodes between patient cases with and without seroma. Since 100 cases are included, the percentages are identical to the absolute numbers.

	Cases with Postoperative Seroma	Cases without Postoperative Seroma	*p*-Value
**Use of a foreign body**			*p* < 0.001
No foreign body Implant (with Mesh) Expander	43 7 2	24 21 3	
**Axillary intervention**			*p* = 0.137
None	1	3	
SLND	31	22	
ALND	17	8	
TAD	1	7	
SLN + ALND	2	6	
TAD + ALND	2	0	
**Number of removed axillary lymph nodes**			*p* = 0.792
0	0	2	
1	16	11	
2	9	11	
3	3	6	
4–10	7	4	
>10	17	13	
**Number of positive axillary lymph nodes**			*p* = 0.186
0	31	32	
1	5	5	
2	4	3	
3	2	2	
4	2	1	
>4	8	3	

SLND: Sentinel lymph node biopsy. ALND: Axillary lymph node dissection. TAD: Targeted axillary lymph node dissection.

**Table 3:** Binary logistic regression for seroma formation. Binary logistic regression was performed with the factors significant in the correlation analysis. Shown are the corresponding odds ratios (ORs) with 95% confidence interval (CI), significant values are highlighted with an asterisk (*).

	Odds Ratio	95% CI	*p*-Value
**BMI**	0.987	0.874–1.113	0.828
**Age at surgery**	1.054	1.014–1.095	0.007 *
**Grading**	0.318	0.122–0.831	0.019 *
**Ablation weight**	1.002	1.000–1.005	0.109
**Use of foreign body**	1.025	0.627–1.678	0.920
**Tumor stage (pT)**	0.930	0.769–1.125	0.456

## Data Availability

The data presented in this study are available on request from the corresponding author.

## Abbreviations

ALND	Axillary lymph node dissection
BMI	Body-Mass-Index
CI	Confidence interval
CIS	Carcinoma in situ
ER	Estrogen receptor
FISH	Fluorescence in situ hybridisation
HER2	Human epidermal growth factor receptor 2
HR	Hormone receptor
IHC	Immunohistochemistry
NST	Non special type
OR	Odds ratio
PR	Progesterone receptor
SerMa (study)	Seroma formations of the mammary gland in breast cancer patients after mastectomy (study)
SLND	Sentinel lymph node biopsy
TAD	Targeted axillary lymph node dissection
Th	T helper cell
